# Ultrafiltration Process in Disinfection and Advanced Treatment of Tertiary Treated Wastewater

**DOI:** 10.3390/membranes11030221

**Published:** 2021-03-20

**Authors:** Rafał Tytus Bray, Katarzyna Jankowska, Eliza Kulbat, Aneta Łuczkiewicz, Aleksandra Sokołowska

**Affiliations:** Department of Water and Wastewater Technology, Faculty of Civil and Environmental Engineering, Gdansk University of Technology, 11/12 Narutowicza Street, 80-233 Gdansk, Poland; rafal.bray@pg.edu.pl (R.T.B.); ekul@pg.edu.pl (E.K.); ansob@pg.edu.pl (A.Ł.); aluk@pg.edu.pl (A.S.)

**Keywords:** membranes, indicator bacteria, microscopic methods

## Abstract

The paper presents the results of research on the use of ultrafiltration, using membranes of 200 and 400 kDa separation, for disinfection of municipal treated wastewater. The research was conducted on a fractional technical scale using real municipal treated wastewater from two large wastewater treatment plants treating most of the wastewater over the one-million polycentric Gdańsk agglomeration (1.2 million inhabitants). UF 200 kDa and UF 400 kDa processes enabled further improvement of the physical and chemical parameters of treated wastewater. Total phosphorus (to below 0.2 mg/L–UF 200 kDa, 0.13 mg/L–UF 400 kDa) and turbid substances (to below 0.2 mg/L, both membranes) were removed in the highest degree. COD was reduced efficiently (to below 25.6 mgO_2_/L–UF 200 kDa, 26.8 mgO_2_/L–UF 400 kDa), while total nitrogen was removed to a small extent (to 7.12 mg/L–UF 200 kDa and 5.7 mg/L–UF 400 kDa. Based on the reduction of indicator bacteria; *fecal coliforms* including *E. coli* (FC) and *fecal enterococci* (FE) it was found that the ultrafiltration is an effective method of disinfection. Not much indicator bacterial were observed in the permeate after processes (UF 200 kDa; FC—5 CFU/L; FE—1 CFU/L and UF 400 kDa; FC—70 CFU/L; FE—10 CFU/L. However, microscopic analysis of prokaryotic cells and virus particles showed their presence after the application of both membrane types; TCN 3.0 × 10^2^ cells/mL–UF 200 kDa, 5.0 × 10^3^ cells/mL–UF 400 kDa, VP 1.0 × 10^5^/mL. The presence of potentially pathogenic, highly infectious virus particles means that ultrafiltration cannot be considered a sufficient disinfection method for treated wastewater diverted for reuse or discharged from high load wastewater treatment plants to recreational areas. For full microbiological safety it would be advisable to apply an additional disinfection method (e.g., ozonation).

## 1. Introduction

The significant microbiological contamination of treated wastewater, shown in numerous works [1,2,3,4] draws the attention of sewage treatment plant operators to the disinfection processes [5,6,7,8,9,10,11]. Treated wastewater contain not only eggs of gastrointestinal parasites and pathogenic bacteria, but also various types of fecal bacteria with exhibiting of multi-resistance for commonly used antibiotics [12,13]. Among them, bacterial strains that carry R plasmids and integrons can contaminate receiver waters or crops and transfer intestinal bacterial resistance traits into consumer organisms [14,15]. Therefore, aquatic ecosystems can become reservoirs of antibiotic resistance genes [16]. Moreover, other research conducted on a model [17] and technical scale [18,19,20] indicate a positive selection of bacteria which show the features of multi-resistance, by wastewater treatment processes with the activated sludge method. Another significant threat are pathogenic viruses, commonly found in raw and treated sewage [21,22,23] as well as in the environment receiving wastewater or other pollutants of fecal origin [23,24]. Their removal is particularly important in the context of the epidemiological threat associated with the current Covid-19 pandemic [25,26,27], as well as dissemination of other viral diseases [23,24,28].

Alternative methods of treated wastewater disinfection applied on technical scale include chemical (with the use of chlorine, ozone, peracetic acid) and physical methods (with the use of UV radiation, membranes) and they differ in the efficiency of removing microorganisms [29,30,31]. For example, chlorine and peracetic acid are effective disinfectants against bacteria of intestinal origin, while they are ineffective for viruses, bacterial spores and protozoan cysts, though the important advantage of peracetic acid is its low potential to form harmful by-products and ease of degradation into harmless products (acetic acid and oxygen) [6,30].

Although irradiation with UV rays effectively removes viruses, bacterial spores and protozoan cysts, a serious disadvantage of this method is the possibility of bacterial re-growth due to reactivation effect (in the light or in the dark) and that the necessary dose largely depends on the physico-chemical characteristics of the sewage [32,33]. Wastewater disinfection with ozone is often recognized as an equivalent to the UV-radiation method, but requires more funding [34]. It has a positive effect on the smell and color of sewage, but it increases the concentration of biodegradable organic substances [30]. In order to increase the effectiveness of the disinfection process, it is often necessary to precede this process by filtration treated wastewater [35]. Another significant aspect, considered when choosing the appropriate disinfection method, is the potential for the formation of toxic, mutagenic and/or carcinogenic by-products, e.g., trihalomethanes—usually formed during chlorination of sewage [36,37,38,39,40]. That is why physical methods using low-pressure processes (like microfiltration and ultrafiltration) and high-pressure processes (like reverse osmosis and nanofiltration), which avoid the introduction of harmful by-products into the environment, are becoming more and more popular. In order to reduce costs and waste production, substitutes for relatively expensive synthetic materials are increasingly being sought in membrane technologies. One solution could be the nanocellulose-enabled membrane technology [41,42] including its modifications [43,44,45].

Increasingly the membrane techniques are successfully used in water treatment technology, including large drinking water treatment installations, but in wastewater treatment they are used rather in the treatment of relatively small amounts of sewage, mainly industrial ones. The separation of microorganisms from water or sewage in membrane processes takes place thanks to the sieve mechanism, and the effectiveness of microorganisms retention is determined by their size and pore size of the membrane [31]. For or bacteria, the smallest of which are 0.2–0.3 µm, the use of ultrafiltration, where the pores of the membranes are smaller than 0.1 µm, should guarantee a 100% efficiency of their retention.

In the case of drinking water treatment, despite the interesting effects of retaining bacteria and even viruses, it is emphasized that no membrane filtration system can be considered as an absolute barrier for all microorganisms [31]. This is primarily due to imperfections of membranes and membrane modules and the possibility of secondary bacterial growth in water after passing through the membrane [31]. In addition, the cells of microorganisms can penetrate the pores of the membrane with diameters much smaller than the dimensions of the cells themselves, due to pressure deformation [31,46]. If membrane techniques are used to disinfect treated wastewater discharged into the environment, this imperfection of membranes is not so significant as it is not necessary to obtain a full guarantee of retention of all microorganisms. An additional advantage of membrane techniques in this case is the possibility of improving the physico-chemical quality of the wastewater before it enters the environment [47].

The aim of this study is to evaluate the effectiveness of ultrafiltration processes in the disinfection of treated municipal wastewater, carried out under technical and operational conditions, and to assess the impact of these processes on the physico-chemical properties of treated wastewater discharged to surface waters.

The main novelty of the presented research was its performance on a technical scale. Its aim was to reproduce, as fully as possible, the technical and operational conditions of membrane ultrafiltration of biologically treated wastewater. The research was carried out in two large municipal wastewater treatment plants, with the use of serially produced membrane modules and ultrafiltration membranes and on real treated wastewater, which is directly discharged from the treatment plant to the receiver.

## 2. Materials and Methods

### 2.1. Study Area

**Figure 1:**
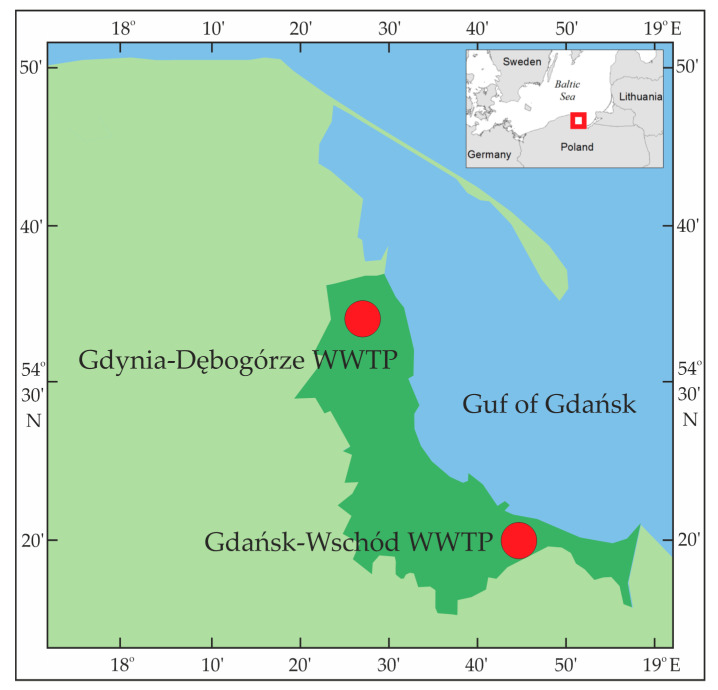
Map with marked wastewater treatment plants on which the research was conducted.

The research was conducted on a fractional technical scale using real municipal treated wastewater from two large wastewater treatment plants (Gdańsk-Wschód and Gdynia-Dębogórze), which treat wastewater from over one million polycentric Gdańsk agglomeration (1.2 million inhabitants [48]), Figure 1. They are the largest municipal WWTPs located in Northern Poland carrying out disposal of wastewater to the Gdańsk Bay (the Baltic Sea). Both facilities are mechanical and biological treatment plants. Gdynia-Dębogórze sewage treatment plant has a biological stage consisting of multiphase bioreactors with activated sludge with a total capacity of 104,000 m^3^ designed in BARDENPHO technology with simultaneous denitrification in the CARROUSEL system, cooperating with secondary radial settling tanks. To maintain phosphorus concentration at an acceptable level, the process of biological dephosphatation is supported by dosing of iron coagulant [49,50]. The Gdańsk-Wschód sewage treatment plant has a biological stage consisting of multiphase activated sludge bioreactors (modified UCT system), cooperating with radial secondary settlers. Periodically an aqueous solution of polyaluminium chloride is used to limit the growth of filamentous bacteria in the activated sludge system [50,51]. Treated wastewater was collected directly from the channels draining the treated wastewater to the Gulf of Gdańsk.

### 2.2. Experimental Setup

Due to the use of the industrial membrane module in the research and testing performed directly at the treatment plant using fresh treated wastewater, the scale of the research can be considered fractionally technical. Membrane filtration was carried out in a mass-produced, industrial, tubular module type B1, produced by PCI Membranes, allowing work with tubular membranes with the MF/UF and RO filtration range [52]. The module was made of stainless steel and contained 18 internal steel perforated pipes, with tubular (12.7 mm in diameter) filter membranes connected in series (1.2 m long) placed inside. The total area of the membrane was 0.9 m^2^. The characteristic feature of B1 type membrane module is the possibility to disassemble it in order to replace used membranes. According to the authors’ own observations, these types of membrane modules may be particularly vulnerable to leakage and the formation of internal seepage between the feed and the permeate. The membrane module was supplied with sewage treated continuously, with cross-flow being used. In cross-flow, the retentate circulated in a closed circuit forced by an additional vortex pump (without an additional feed tank), and the total volume of retentate was about 2.8 dm^3^, which was less than 1.5% of the total volume of permeate. The flow velocity of the retentate inside the membranes was 3.0 m/s. Membrane filtration was carried out at transmembrane pressure from 0.3 to 0.5 MPa. Permeate flux was greater than 50 dm^3^/m^2^/h. There were applied long, multi-hour filtration cycles of 3 h, 5 h and 22 h.

In the tests there were deployed serially produced ultrafiltration membranes made of PVDF by PCI Membranes of two types: with a separation of 200 kDa (FP 200 UF) and with a separation of 400 kDa (XP 201/04/SIN) [52]. In the further part of the text, the following membrane symbols were introduced: UF 200 kDa and UF 400 kDa, respectively. At the end of each filtration cycle and before subsequent tests, the membranes and the entire test system were cleaned as follows: flushing with a powerful stream of tap water (5 min.), cleaning with hot NaOH solution (55–60 °C; pH = 10.5; 15 min), cleaning and disinfection with hot NaOH and NaOCl solution (55–60 °C; pH = 10.5; 15 min.), final rinsing with tap water (5 min.).

### 2.3. Wastewater Sampling

The research was carried out in the spring and summer period. A total of 19 research series were conducted. In the sewage treatment plant in Gdańsk-Wschód there were performed 9 test series, while in the Gdynia-Dębogórze sewage treatment plant the other 10. A single test run was a complete membrane filtration cycle. Due to the use of real wastewater in the research, the composition of treated wastewater used in the tests differed in each series and for each of the treatment plants in terms of physico-chemical and microbiological parameters. Samples for microbiological and physico-chemical tests were collected about 1 h from the beginning of the filtration process (BF) and at the end of the filtration cycle (EF).

Wastewater samples were collected immediately after passing through the filtration module, into sterile 0.5 L polypropylene containers, transported in a dark, cool warehouse (portable refrigerator) and analyzed immediately upon return to the laboratory.

### 2.4. Physico-Chemical Analysis

The suspension concentration was determined by weight, after filtration through nitrocellulose filters with a pore diameter of 1.2 µm (Merck KGaA, Darmstadt, Germany) according to PN-72/C-04559/02, while COD, total nitrogen and total phosphorus were determined using colorimetric methods and a Hach Lange Xion500 spectrophotometer (Dr Lange, GmbH, Homburg, Saarland, Germany) according to the Standard Methods [53].

### 2.5. Microbiological Analysis

#### 2.5.1. Cultivation Methods

Microbiology analyses included cultivation of fecal indicator bacteria (*fecal coliforms* including *E.coli*–FC and *fecal enteroccoci*—FE) and microscopic observations to determine the abundance and characteristics of the prokaryotic microorganisms population present in the wastewater samples. Detection and enumeration fecal indicator bacteria of carried out using membrane filtration of 1 and 10 mL samples via cellulose membrane filter (47 mm diameter, 0.45 μm pore diameter, Whatman, Merck, Germany). *E.coli* bacteria were incubation on mFC agar (Merck KGaA, Darmstadt, Germany) at 44.5 °C for 24 h. Blue colonies were counted as *fecal coliforms* including *E.coli* bacteria (according to ISO 9308-1:2014). Fecal enterococci were cultured on Slanetz-Barteley medium and confirmed on medium with esculin, azide and bile (according to PN-EN IOSO 7899 2: 2002U).

#### 2.5.2. Microscopic Methods

Microbiological parameters such as total prokaryotic cell number (TCN), average prokaryotic cell volume (ACV), prokaryotic biomass (PB) and prokaryotic cell morphotype diversity were determined using direct epifluorescent filter technique (DEFT). Water samples (50 mL) were fixed with buffered formalin to a final concentration of 2%. Sub-samples of 1.5 mL were stained with DAPI (4,6-diamidino-2-phenyl-indole) to a final concentration of 1 μg/mL and filtered through a black Nuclepore polycarbonate membrane filter (0.2 μm pore diameter, Whatman, Merck, Germany) [54]. Filters were mounted on a microscopic slide with non-fluorinating oil (Citifluor AF2: Agar Scientific, Stansted, Essex, UK) [55] and stored at −20 °C until analysis.

Microscopic observations were carried out using a Nikon 80i epifluorescence microscope under 1000-fold total useful microscope magnification. A HBO103 W/2 high pressure mercury lamp (Osram, GmbH, Munich, Germany), 330–380 nm excitation filter, 420 nm barrier filter and 400 nm dichroic mirror were used. For each sample, 20 fields of microscope vision with maximum 60 thousand objects were digitalized using Nikon DS-5Mc-U2 high-resolution color digital camera and NIS-Elements BR 3.0 software. Abundance (TCN), size (ACV) and geometric parameters (morphological types) of stained prokaryotic cells were determined with an automatic image analysis system (Multi Scan, v.14.02, CSS, Warsaw, Poland). ) and modification of Świątecki [56]. In order to determine the diversity of cell morphotypes, the frequency of prokaryotic microorganisms morphological forms (cocci, rods and cylindrical curved) was evaluated [57]. Prokaryotic biomass (PB) was obtained from average cell volume using biomass conversion factor of 170 fg C µm^3^ [58].

The possibility of reducing virus particles (VP) in the ultrafiltration process was also evaluated. For this purpose, wastewater subsamples were diluted 500–1000 times with sterile Milli-Q water, deprived of viruses by filtration, and were preserved in buffered formalin (final concentration 2%) and stained with fluorescent dye SYBR^®^Gold (Molecular Probes, Inc., Eugene, Oregon, US) for 15 min in the dark at room temperature. We used a concentrated 1:2500 commercial stock solution in DMSO diluted in MQ water deprived of viruses by filtration [59]. Samples were filtered through 0.02-μm pore-size Al_2_O_3_ membrane filters (Anodisc 25 mm, Whatman, USA) (pressure, ca. 100 mm Hg). Then dried in the dark at room temperature and mounted on glass slides in the presence of antifade solution (Citifluor AF2: Agar Scientific, Stansted, Essex, UK) [55]. Green fluorescent particles in 20 randomly-selected fields were counted (max. 20,000 per sample).

### 2.6. Statistical Analysis

The obtained test results were developed using the procedures of the Excel program (Microsoft Office Standard 2016) and Statistica (13.3). For physico-chemical data (turbidity, chemical oxygen demand (COD), total nitrogen (TN), total phosphorus (TP), basic descriptive statistics were calculated: minimum value, maximum value and arithmetic mean.

For indicator bacteria (*fecal coliforms* including *E.coli* and *fecal enterococci*), total prokaryotic cells number (TPN) and prokaryotic biomass (PB), the results were presented in the form of box plots (“box with a whisker”), in which the minimum and maximum values, 1st and 3rd quartiles and arithmetic mean were marked. Drawings have been edited in CorelDRAW 2019. The statistical procedures for image analysis in microscopy are described above.

## 3. Results and Discussion

### 3.1. Physico-Chemical Analysis

The process of ultrafiltration of treated wastewater resulted in significant changes in its physico-chemical quality. The best results were obtained for turbidity, which was reduced to trace levels for both types of membranes. The permeate turbidity, regardless of the turbidity of the sewage supplied to the system and the method and parameters of ultrafiltration, was low and for membranes with 200 kDa and 400 kDa separation it did not exceed appropriately 0.02 NTU and 0.1 NTU, Table 1.

**Figure 2:**
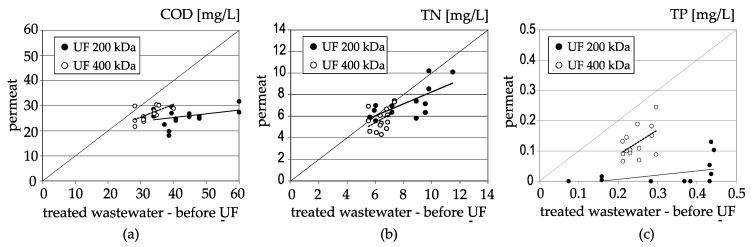
Concentrations of selected physico-chemical parameters: (**a**)—chemical oxygen demand—COD; (**b**)—total nitrogen—TN; (**c**)—total phosphorus–TP, in the permeate after ultrafiltration in relation to their concentration in treated sewage supplied to the filtration system.

Slightly worse and unstable effects were obtained in relation to the chemical oxygen demand (COD) value, while overall better results were obtained for membranes with 200 kDa separation. In the case of membranes with 200 kDa separation, the reduction of the COD parameter ranged from 15.3% to 54.3% (average 37.8%), and the COD value in the permeate was from 18.1 to 31.7 mg O_2_/L. In the case of membranes with 400 kDa separation, the COD decreased from 0% to 27.8% (on average 17.6%), and the permeate COD value fluctuated from 21.7 to 30.4 mg O_2_/L Table 1, Figure 2a.

**Figure 3:**
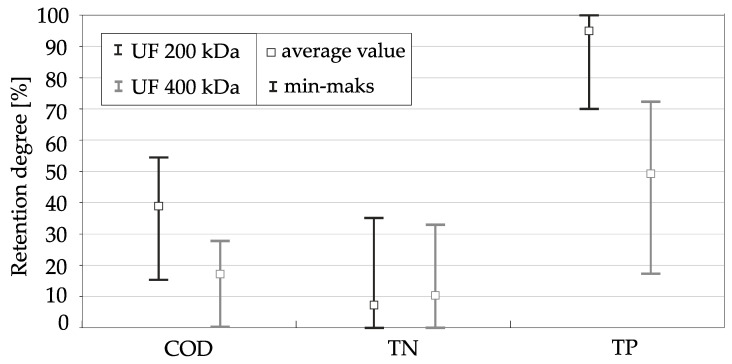
Comparison of retention coefficients of selected chemical parameters (chemical oxygen demand—COD; total nitrogen—TN; total phosphorus–TP) of biologically treated wastewater subjected to membrane filtration with the use of ultrafiltration membranes with separation of 200 kDa and 400 kDa.

Total nitrogen (TN) was removed only to a small degree for both membranes. The average retention factor was 8.4% for membranes with 200 kDa separation and 10.9% for membranes with 400 kDa separation, Table 1, Figure 2b. It was also the only one of the tested components that was removed slightly better with the use of membranes with 400 kDa separation, Table 1, Figure 3. It was probably due to high proportion of dissolved nitrogen forms in total nitrogen [50,60] provided the measurements of primary and secondary effluent total nitrogen and component fractionation in wastewater treatment plants in Gdynia and Gdańsk and proved that the contributions of dissolved organic nitrogen in total organic nitrogen was even 45%.

Among the chemical parameters, the reduction of total phosphorus (TP) concentration was obtained in high degree, and better results were obtained for membranes with 200 kDa separation, for which total phosphorus was removed almost completely. The total phosphorus retention coefficient ranged from 70% to 100% (average 95%), and its concentration in the permeate did not exceed 0.1 mgP/L, while in over 2/3 of the measurements it was lower than 0.01 mgP/L. In the case of membranes with the separation of 400 kDa, the effects of total phosphorus removal were also considerable and its concentration after ultrafiltration decreased by half on average. The retention coefficient varied from 17.2% to 72.3% (average 49.4%) Table 1, Figure 2c. The concentration of total phosphorus in the permeate in the vast majority of samples did not exceed 0.2 mgP/L, while in almost half of the measurements it was lower than 0.1 mgP/L. The noticeable effects of total phosphorus removal in the ultrafiltration process can be explained by the presence of a significant part of it in an undissolved form in treated wastewater, which was the result of using both the biological dephosphatation process and the use of coagulants in sewage treatment plants [61,62].

For the membranes with 200 kDa and 400 kDa separation, deterioration of the filtration effects was observed at the end of the filtration cycle, with greater deterioration of the purification effects for the UF 400 kDa membranes Table 1. Only in the case of total phosphorus, the duration of the filtration cycle did not affect the efficiency of its removal. The deterioration of the effects after a longer duration of microfiltration occurred most likely due to the homogenization of the suspension and the progressive decomposition of organic matter, mainly from the suspension accumulated in the condensate, and the release of dissolved or colloidal organic compounds.

Comparing the results for both types of membranes, the use of ultrafiltration membranes with lower separation allows for better wastewater treatment effects, Table 1, Figure 3.

Using both types of membranes, there were removed mainly undissolved impurities fractions, usually related to the suspension. Colloidal fractions were partially removed and most likely these fractions were responsible for the differences in the removal of individual impurities. The colloidal fractions were removed to a greater extent using membranes with a smaller separation (200 kDa). Solutes remained in the permeate after ultrafiltration, regardless of the pore size of the membranes.

In general, the quality of the permeate and the degree of removal of individual components of the sewage was determined primarily by the form of their occurrence in the sewage. It was observed that the components/parameters whose concentration correlated with the amount of suspended solids in the effluent from the treatment plant (turbidity, total phosphorus, COD) were removed better from the wastewater in the ultrafiltration process than components where no such correlation was found (mainly total nitrogen). Thus, determining the level of dissolved, colloidal and undissolved fractions of individual indicators of treated wastewater may be important in forecasting the effectiveness of their removal using membrane techniques [60].

### 3.2. Reduction of Indicator Bacteria (Fecal Coliforms, including E.coli–FC and Fecal Enteroccoci—FE)

After ultrafiltration, significant changes in the physico-chemical quality of treated wastewater were noted. Apart from that, significant elimination of indicator bacteria, as well as a change in the number and structure of prokaryotic community were observed. It should be noted here that because the research used treated wastewater from a full-scale technological process in two different treatment plants, the number of indicator bacteria was different in successive research series. In case of a series where UF 200 kDa separation membranes were used, the number of E. coli bacteria in the pre-filtration wastewater (TW) was in the range 1.8–3.0 × 10^5^ CFU/L. For the series where UF 400 kDa separation membranes were used, the *fecal coliforms* (including *E.coli*)–FC, count ranged from 1.0–6.0 × 10^5^ CFU/L. For *fecal enterococci*–FE, these were values respectively: UF 200 kDa from 8.9 × 10^4^ to 4.8 × 10^5^ CFU/L and UF 400 kDa from 1.0 to 4.0 × 10^5^ CFU/L. Despite differences, for both groups of indicator bacteria, these were values in accordance with the results obtained previously on these wastewater treatment plants [18,19] and those reported in other studies [11,63].

The presence of a significant number of indicator bacteria in treated wastewater confirms observations that, although conventional treatment processes (primary and secondary treatments) remove up to 95–99% of some micro-organisms, the properties of treated wastewater make it unsuitable for direct re-use due to the presence of potentially high concentrations of pathogenic micro-organisms [64]. Moreover, due to the significant wastewater flow of treated wastewater discharged from large urban wastewater treatment plants, such as those discussed in this study (population equivalent Gdańsk Wschód—840,200 PE, Gdynia Dębogórze—440,000 PE) [65], the load of pathogenic bacteria brought into the receiver (in this case, these are coastal areas of the Baltic Sea, of great recreational value) may pose a threat to public health and the environment [66,67]. Therefore, the need to disinfect waste water becomes a necessity [5,11,68,69,70]. Membrane filtration is one of the alternatives to commonly used methods such as chlorination, UV or ozone. Filtration membranes were used in the research, whose pore diameter should retain procaryotic cells, due to their size. Actually, the retention efficiency for indicator bacteria was high on both types of membranes, both during ultrafiltration with UF 200 kDa and UF 400 kDa membranes. The differences between types of membranes were observed mainly at the end of the filtration cycle.

**Figure 4:**
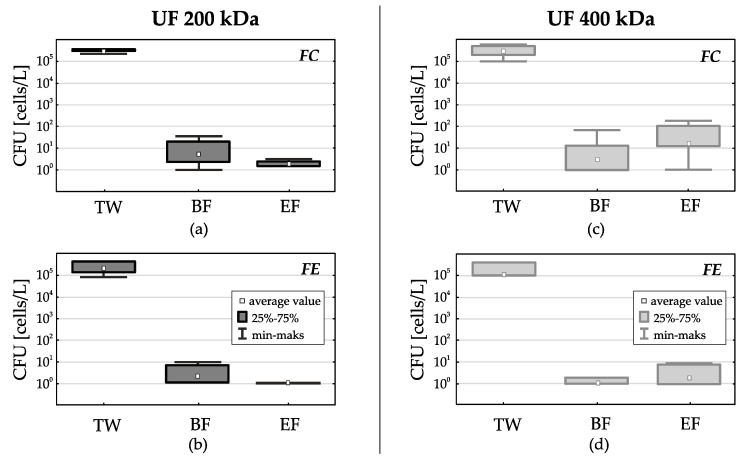
Comparison of the indicator bacteria number in treated wastewater (TW) subjected to membrane filtration with the use of ultrafiltration membranes with separation of 200 kDa and 400 kDa at the beginning of the filtration cycle (BF) and at the end of the filtration cycle (EF); (**a**)—FC, 200 kDa; (**b**)—FE, 200 kDa; (**c**)—FC, 400 kDa; (**d**)—FE, 400 kDa.

The use of membranes with UF 200 kDa separation has resulted in a significant reduction of the *fecal coliforms* (including *E.coli*)–FC. At the beginning of the filter cycle (BF) their average number decreased by 4 log (to below 20 CFU/L) and at the end of the filter cycle (EF) their average number was 5 CFU/L (Figure 4a). For *fecal enterococci*–FE, at the beginning of the filtration cycle (BF) their average number decreased by 5 logs (below 5 CFU/L) and at the end of the filtration cycle (EF) their average number did not exceed 1 CFU/L (Figure 4b).

Other dependencies were found when using membranes with UF 400 kDa separation. Here too, at the beginning of the filter cycle (BF) a significant reduction of indicator bacteria was noted. For *FC* the average reduction was 4 log (below 70 CFU/L), for *FE*—5 log (below 1 CFU/L). However, at the end of the filter cycle (EF), the number of indicator bacteria in the permeate increased again. For *FC* the average number increased to 1.8 × 102 CFU/L, (Figure 4c) and for FE to 10 CFU/L (Figure 4d). The increase in the number of bacteria in the permeate towards the end of the filter cycle was probably due to a significant increase in the number of bacteria in the condensate [31].

### 3.3. Reduction of Single Prokaryotic Cells Determined by Microscopic Method

Apart from the indicator bacteria reduction assessment, also another microbiological parameter based on direct microscopic analysis were estimated: total prokaryotic cells number (TCN), prokaryotic biomass (PB) and the percentage share of rods, cocci and curved shaped cells divided into five volume size classes. The combination of fluorescent microscopy techniques and computer image analysis methods allows to obtain fully reproducible and statistically reliable results [71,72,73,74]. In the research presented in this work the automatic image analysis system combined with statistical analysis developed by Świątecki was used [56]. This solution has been successfully applied by the authors of this work in studies of various types of environments [75,76,77,78].

**Figure 5:**
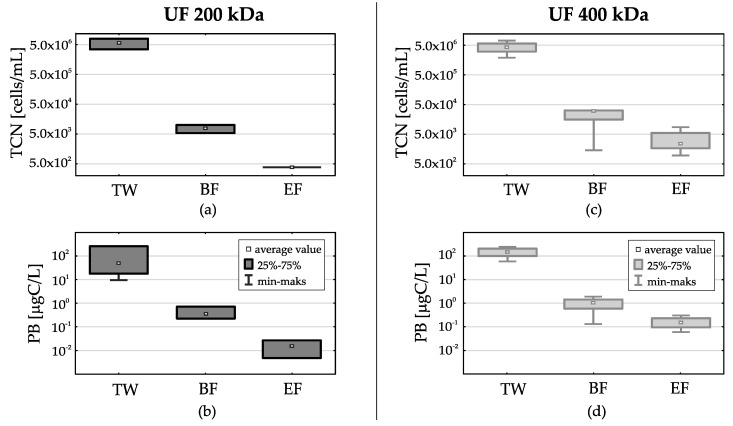
Comparison of the total prokaryotic cells number (TCN) and the prokaryotic biomass (PB) in treated wastewater (TW) and subjected to membrane filtration with the use of ultrafiltration membranes with separation of 200 kDa and 400 kDa, at the beginning of the filtration cycle (BF) and at the end of the filtration cycle (EF); (**a**)—TCN, 200 kDa; (**b**)—PB, 200 kDa; (**c**)—TCN, 400 kDa; (**d**)—PB, 400 kDa.

Filtration on both types of membranes has considerably reduced the total prokaryotic cells number (TCN) and prokaryotic biomass (PB). In treated wastewater the average value was for TCN 5.0 × 10^6^ cells/mL and for PB 132 µgC/L, which were similar to the values previously quoted by the authors [79]. At the beginning of the filtration cycle (BF) for UF 200 kDa the average value for TCN and PB dropped by 3 log, and for UF 400 kDa decreased by 2.5 log. At the end of the filtration cycle (EF) a further TCN and PB drop was noted for both membrane types. For the UF 200 kDa membrane average value TCN was below 300 cells/mL and PB 0.02 µgC/L. However, for the UF 400 kDa membrane the reduction was not so evident—in some series of tests it exceeded TCN 5.0 × 10^3^ cells/mL and PB 0.37 µgC/L) (Figure 5a,c).

**Figure 6:**
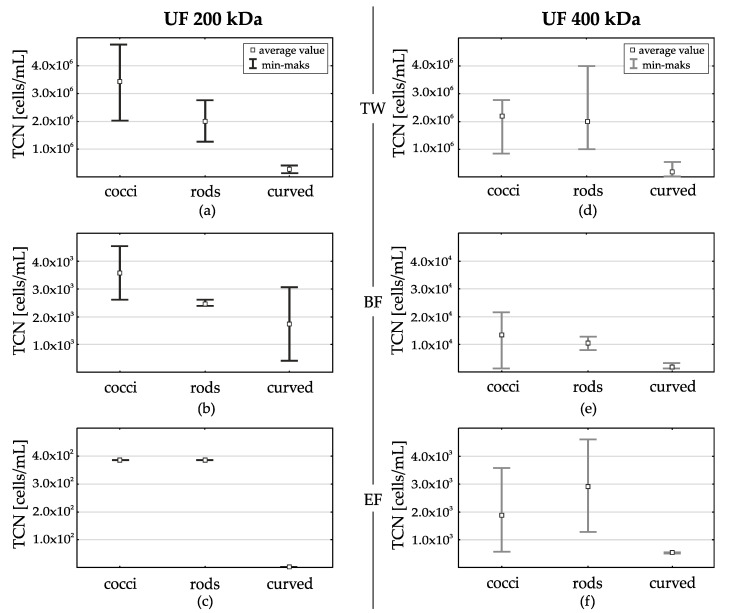
Comparison of the cells number of differently shaped (cocci, rods and curved), in treated wastewater (TW) and subjected filtration using membranes UF 200 kDa and UF 400 kDa [(**a**,**d**), respectively], at the beginning of the filtration cycle (BF) [(**b**,**e**) respectively] and at the end of the filtration cycle (EF) [(**c**,**f**) respectively].

The occurrence of procaryotic community cells of different shapes (cocci, rods, curved) was also analyzed. In treated wastewater, due to different parameters of wastewater in successive research series, similarly to the number of indicator bacteria, differences in the number of procaryotic cells in particular shapes were also noted. It was generally noted, that cocci dominated in the treated wastewater. (average value 2.2 × 10^6^—3.4 × 10^6^ TCN/mL) Rods were less numerous in all series (a.v. 1.9 × 10^6^ TCN/mL). The least numerous were curved (a.v. 1.8 × 10^5^–2.7 × 10^5^ TCN/mL) (Figure 6a,d).

After the filtration process, with the use of both types of UF membranes, a significant decrease in the number of cells of all shapes was noticed. For UF 200 kDa after the first stage it was a decrease of 3 log for cocci and rods and 2 log for curved. At the end of the filtration cycle only cocci and rods (a.v. 3.8 × 10^2^ TCN/mL) were found. For UF 400 kDa in the first stage this decrease was slightly smaller than after filtering by UF 200 kDa—for cocci, rods and curved by 2 log. At the end of the filtration cycle a further decrease in the number of cells was observed, but more cells were observed than after filtration with UF 200 kDa (Figure 6b,e).

Average number of cells for: cocci—1.8 × 10^3^ TCN/mL, rods—2.8 × 10^3^ TCN/mL and curved 5.5 × 10^2^ TCN/mL. The particularly high number of rods recorded in the permeate after UF 400 kDa filtration seems interesting (Figure 6c,f). It was noted that it is consistent with the results for *FC* (*fecal coliforms* including *E.coli*), which also increased at the end of the filtration process with UF 400 kDa. It is possible that the reason why bacilli penetrated the membranes to a greater extent than cells of other shapes is because of their elongated shape (much smaller dimension in one axis).

**Figure 7:**
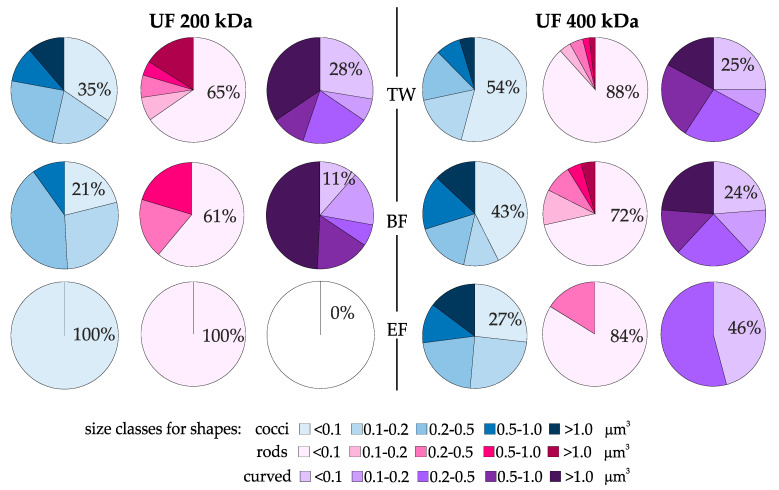
Comparison of the percentage share of cocci, rods and curved, with respect to five bio-volume classes in treated wastewater (TW) subjected to membrane filtration with the use of ultrafiltration membranes with separation of 200 kDa and 400 kDa at the beginning of the filtration cycle (BF) and at the end of the filtration cycle (EF).

Differences in the total reduction of prokaryotic cells may result not only from their different shapes but also from their different sizes [68]. Therefore, the percentage share of particular cell shapes (rods, cocci and curved) was also analyzed, taking into account their size (division into five bio-volume classes) (Figure 7). Clear differences between permeate coming from different membranes were observed. For UF 200 kDa membrane a clear reduction of larger rods and cocci was noted. At the end of the filtration cycle (EF) only the smallest cells (<0.1 µm^3^) were recorded in the permeate. Larger forms of curved cells appeared more numerous at the beginning of the filtration cycle (BF), and then they were not recorded at all. Although the UF 400 kDa membrane clearly decreased TCN (2.5 log—Figure 5c) at the beginning of the filtration cycle (BF), cells with a larger volume also appeared in the permeate, even at the end of the filtration cycle (EF).

Despite significant reduction of prokaryotic cells number due to the ultrafiltration process, their presence in the permeate can be surprising. The pore size of UF membranes is much smaller than the length or width of individual cells, therefore theoretically even single cells should not be observed in the permeate. Nevertheless, the presence of prokaryotic cells in the permeate after UF is indicated by many authors [31,46,80,81]. Membrane pores smaller that the cell sizes could be penetrated by microorganisms due to their pressure deformation [46,80]. UF membranes are also not a sufficient barrier to ultramicrobacteria [82]. In addition, especially in the case of technical installations, discontinuities and damage to the membrane skin layer as well as possible leaks in the membrane module are indicated [31].

### 3.4. Reduction of Virus Particles (VP)

**Figure 8:**
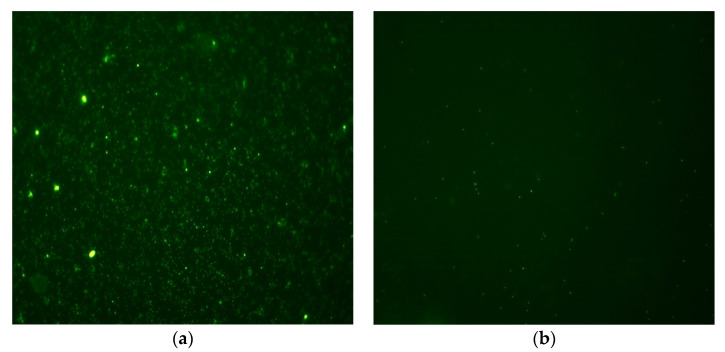
Virus particles (VP) stained with SYBR^®^Gold fluorochrome (**a**) in treated wastewater; (**b**) after filtration with a UF 400 kD membrane at the end of filtration (EF). Nikon Eclipse 90i microscope and digital camera Nikon DS-5Mc-U2 were used. Pictures done in Gdansk University of Technology, Faculty of Civil and Environmental Engineering, Department of Water and Wastewater Technology, photo: K. Jankowska.

As mentioned before, virus particles (VP) present in the wastewater may also pose an epidemiological risk [21,22,23,27]. Viruses are typically too small (20–200 nm) to be observed and counted under a light microscope. However, after being captured on an alumina filter (pore diameter 0.02-μm–Anodisc 25 mm, Whatman, USA) and stained with the sensitive DNA dye SYBR Green I [83,84] or SYBR Gold [59,85], they can be visualized and counted on an epifluorescence microscope. However, when stained with Sybr Green I or SYBR Gold, they can be observed under epifluorescence microscope. Based on that method, the potential of VP reduction by ultrafiltration was tested in this study (Figure 8). The UF200 kDa membrane was selected for VP reduction tests.

In our study, the average number of virus particles in treated wastewater was equal to 7.8 × 10^9^/mL which is in agreement with other studies [21]. At the end of filtration cycle (EF) the amount of VP decreased by more than 4 log_10_ (average: 1.0 × 10^5^/mL), which corresponds to 99.9% reduction requirement (EPA US). It should be noted that many of the virus particles (VP) present in treated wastewater are bacteriophages or non-pathogenic forms. For example, in a study carried out in Japan, the number of colifags in treated wastewater was estimated for 10^3^–10^4^ copies/mL [86]. However, a study conducted in the UK on human Norovirus (NoV), which is responsible for millions of gastrointestinal diseases in developed countries, the number of copies of the virus after secondary wastewater treatment reached 1.0 × 10^6^ copies/mL [23]. Moreover, given the high infectivity of pathogenic virus particles and the very significant volume of treated wastewater discharged to the environment by the treatment plants analysed in these studies (Gdańsk Wschód—33,930,000 m^3^/year; Gdynia Debogórze—20,182,310 m^3^/year) [65], the VP reduction achieved cannot be considered sufficient. The discharge of such wastewater into the environment in recreational areas may pose a serious epidemiological risk [24], especially in the context of the threat posed by the current Covid-19 pandemic [25,26,27].

In the context of the presented results, it should be emphasized that technical membranes and membrane modules produced serially were used in this study. Applied modules allow for module disassembly and membrane replacement. Authors’ observations have shown that such modules may be particularly vulnerable to leaks. The installation of the membranes in a single module required 36 gaskets, and their installation posed some problems. Furthermore, it was not possible to carry out a system leak test after the membranes installation. Therefore, it cannot be excluded that the presence of prokaryotic cells and virus particles in the permeate was the result of the leakage of the membrane module and the imperfections of the membranes themselves. This was also noted in the case of reverse osmosis membranes [47]. Moreover, the possibility of secondary bacterial growth in water after membrane filtration cannot be rejected [31,46]. Therefore, to ensure full epidemiological safety, the ultrafiltration process should be combined with other disinfection technologies—e.g., UV or ozone application [10,23].

## 4. Conclusions

The conducted studies of treated wastewater ultrafiltration allow for the following conclusions:

The ultrafiltration process enables further improvement of the physico-chemical parameters of treated wastewater. Suspension, permeability and total phosphorus were removed the best up to a trace level in most samples. High phosphorus removal efficiency is important because of the ability to remove phosphorus in biological wastewater treatment is limited, especially in small treatment works [87,88] and, moreover, the eutrophication of the water bodies is one of the most important environmental problems.

Good results were also obtained in the removal of COD. On the other hand, total nitrogen was removed to a small extent. The use of membranes with lower separation allows for better sewage treatment results. Most likely, the colloidal fractions were responsible for the differences in the removal of individual pollutants.

The permeate quality and the degree of removal of individual wastewater components was determined primarily by the form of their presence in the wastewater. Thus, determining the level of dissolved, colloidal and undissolved fractions of individual indicators of treated wastewater may be of significant importance in forecasting the effectiveness of their removal using membrane techniques.

The use of ultrafiltration resulted in a significant reduction in the number of indicator bacteria in the treated wastewater. For both separation membranes (200 kDa and 400 kDa), a reduction in the number of *fecal coliforms* (including *E.coli*) by 4 log and *fecal enterococci* by 5 log was achieved.

However, the research showed, that in technical and operational conditions the disinfection effects of biologically treated wastewater using ultrafiltration are worse than expected. Despite the use of membranes with pore sizes much smaller than the size of the bacteria, bacterial cells are still observed in the permeate. Most likely this is due to leaking diaphragm modules and/or damaged diaphragms.

Single cells of indicator bacteria were observed in the permeate, slightly more numerous when UF 400 kDa membrane was used. Microscopic analysis also confirmed the presence of prokaryotic cells in the permeate after the ultrafiltration, regardless the membrane types. The membrane UF 200 kDa was more effective in retaining larger cells.

It was observed that after passing through the ultrafiltration membrane, regardless of the significant decrease in the total number of bacteria, the proportions of prokaryotic cells grouped according to their morphology and size changed. In general, the permeate had a higher percentage of smaller and more compact cells (cocci, rods) than the effluent before filtration. This partially selective membrane action was more pronounced during filtration through more compact membranes (200 kDa) and at the end of the filtration cycle.

On the basis of the tests carried out, it is not possible to directly determine by which routes microorganisms enter the permeate. Whether directly through the membrane structure or through leaks in the membrane module. However, the fact that for a less compact membrane, the retention efficiency of prokaryotic cells was lower and that there was less selection due to their size and shape may indicate that at least some bacteria enter through the membrane structure, through the membrane pores or discontinuities in the epidermal layer.

Average number of virus particles were reduced over 4 log, however, due to the high infectivity of pathogenic virus particles potentially present in treated wastewater that are discharged to recreational areas, it cannot be considered sufficient.

Ultimately, on the basis of the studies carried out, we conclude that ultrafiltration can be regarded as an effective method of wastewater disinfection. However, using it on a technical scale requires integrated systems.

Further research should be carried out in order to identify the causes of worse ultrafiltration effects in technical membrane systems, to select the places, where microorganisms enter the permeate and to determine the scale of this phenomenon.

## Figures and Tables

**Table 1:** Physico-chemical parameters of treated wastewater before and after ultrafiltration.

	Turbidity		Chemical Oxygen Demand (COD)		Total Nitrogen (TN)		Total Phosphorus (TP)	
	NTU	Retention %	mg O_2_/L	Retention %	mg/L	Retention %	mg/L	Retention %
Treated wastewater—Gdańsk-Wschód WWTP								
Average	3.99		41.9		8.2		0.32	
Max	5.35		60.0		11.5		0.44	
Min	1.77		33.9		5.6		0.07	
Treated wastewater—the Gdynia-Dębogórze WWTP								
Average	1.89		32.8		6.38		0.24	
Max	2.20		28.2		5.51		0.21	
Min	1.69		40.0		7.36		0.30	
Permeate—membranes with 200 kDa separation								
Average	<0.02	99.5	25.6	37.8	7.12	8.4	0.02	94.4
Average at the BF ^1^	<0.02	99.5	25.4	37.7	7.1	10.0	0.02	95.1
Average at the EF ^2^	<0.02	99.5	25.8	38.0	7.2	6.3	0.03	93.6
Max	<0.02	99.5	31.7	15.3	10.2	34.8	0.10	100
Min	<0.02	99.5	18.1	54.3	5.6	0.0	0.00	70.2
Permeate—membranes with 400 kDa separation								
Average	0.02	98.9	26.8	17.6	5.7	10.9	0.13	49.4
Average at the BF ^1^	0.02	98.6	26.1	20.3	5.5	14.0	0.12	49.7
Average at the EF ^2^	0.02	99.1	27.7	14.0	6.1	6.6	0.13	48.9
Max	0.09	100	30.4	27.8	4.31	33.0	0.25	72.3
Min	0.00	95.2	21.7	0.0	7.44	0.0	0.07	17.2

^1^ BF—beginning of the filtration cycle; ^2^ EF—end of the filtration cycle.

## Data Availability

Data has not been placed in publicly archived datasets.

## References

[1] Stampi S., De Luca G., Zanetti F. (2001). Evaluation of the efficiency of peracetic acid in the disinfection of sewage effluents. J. Appl. Microbiol..

[2] Koivunen J., Heinonen-Tanski H. (2005). Peracetic acid (PAA) disinfection of primary, secondary and tertiary treated municipal wastewaters. Water Res..

[3] Olańczuk-Neyman K., Stosik-Fleszar H., Mikołajski S. (2001). Evaluation of Indicator Bacteria Removal in Wastewater Treatment Processes. Polish J. Environ. Stud..

[4] Mansfeldt C., Deiner K., Mächler E., Fenner K., Eggen R.I.L., Stamm C., Schönenberger U., Walser C., Altermatt F. (2020). Microbial community shifts in streams receiving treated wastewater effluent. Sci. Total Environ..

[5] Amin M.M., Hashemi H., Bovini A.M., Hung Y.T. (2013). A review on wastewater disinfection. Int. J. Environ. Health Eng..

[6] Bray R., Jankowska K., Kowal P., Kulbat E., Łuczkiewicz A., Olańczuk-Neyman K., Quant B., Sokołowska A., Olańczuk-Neyman K., Quant B. (2015). Dezynfekcja Ścieków.

[7] Azis K., Vardalachakis C., Ntougias S., Melidis P. (2017). Microbiological and physicochemical evaluation of the effluent quality in a membrane bioreactor system to meet the legislative limits for wastewater reuse. Water Sci. Technol..

[8] Alexander J., Kirchen S., Andreas D., Thomas J., Hiller C., Schwartz T. (2018). Live-dead discrimination analysis, qPCR assessment for opportunistic pathogens, and population analysis at ozone wastewater treatment. Environ. Pollut..

[9] Arola K., Van der Bruggen B., Mänttäri M., Kallioinen M. (2019). Treatment options for nanofiltration and reverse osmosis concentrates from municipal wastewater treatment: A review. Crit. Rev. Environ. Sci. Technol..

[10] Hembach N., Alexander J., Hiller C., Wieland A., Schwartz T. (2019). Dissemination prevention of antibiotic resistant and facultative pathogenic bacteria by ultrafiltration and ozone treatment at an urban wastewater treatment plant. Sci. Rep..

[11] Cui Q., Liu H., Yang H.W., Lu Y., Chen Z., Hu H.Y. (2020). Bacterial removal performance and community changes during advanced treatment process: A case study at a full-scale water reclamation plant. Sci. Total Environ..

[12] Inyinbor A.A., Bello O.S., Fadiji A.E., Inyinbor H.E. (2018). Threats from antibiotics: A serious environmental concern. J. Environ. Chem. Eng..

[13] Bondarczuk K., Piotrowska-Seget Z. (2019). Microbial diversity and antibiotic resistance in a final effluent-receiving lake. Sci. Total Environ..

[14] Kotlarska E., Łuczkiewicz A., Pisowacka M., Burzyński A. (2015). Antibiotic resistance and prevalence of class 1 and 2 integrons in Escherichia coli isolated from two wastewater treatment plants, and their receiving waters (Gulf of Gdansk, Baltic Sea, Poland). Environ. Sci. Pollut. Res..

[15] Fiorentino A., Di A., Eckert E.M., Rizzo L., Fontaneto D., Yang Y., Corno G. (2019). Impact of industrial wastewater on the dynamics of antibiotic resistance genes in a full-scale urban wastewater treatment plant. Sci. Total Environ..

[16] Newton R.J., Mcclary J.S. (2019). The flux and impact of wastewater infrastructure microorganisms on human and ecosystem health. Curr. Opin. Biotechnol..

[17] Łuczkiewicz A., Fudala-Ksiązek S., Jankowska K., Quant B., Olańczuk-Neyman K. (2010). Diversity of fecal coliforms and their antimicrobial resistance patterns in wastewater treatment model plant. Water Sci. Technol..

[18] Łuczkiewicz A., Jankowska K., Fudala-Książek S., Olańczuk-Neyman K. (2010). Antimicrobial resistance of fecal indicators in municipal wastewater treatment plant. Water Res..

[19] Luczkiewicz A., Jankowska K., Bray R., Kulbat E., Quant B., Sokolowska A., Olańczuk-Neyman K. (2011). Antimicrobial resistance of fecal indicators in disinfected wastewater. Water Sci. Technol..

[20] Łuczkiewicz A., Felis E., Ziembinska A., Gnida A., Kotlarska E., Olańczuk-Neyman K., Surmacz-Gorska J. (2013). Resistance of Escherichia coli and Enterococcus spp. to selected antimicrobial agents present in municipal wastewater. J. Water Health.

[21] Wu Q., Liu W.T. (2009). Determination of virus abundance, diversity and distribution in a municipal wastewater treatment plant. Water Res..

[22] Brown M.R., Camézuli S., Davenport R.J., Petelenz-Kurdziel E., Øvreås L., Curtis T.P. (2015). Flow cytometric quantification of viruses in activated sludge. Water Res..

[23] Campos C.J.A., Avant J., Lowther J., Till D., Lees D.N. (2016). Human norovirus in untreated sewage and effluents from primary, secondary and tertiary treatment processes. Water Res..

[24] Vergara G.G.R.V., Rose J.B., Gin K.Y.H. (2016). Risk assessment of noroviruses and human adenoviruses in recreational surface waters. Water Res..

[25] Rimoldi S.G., Stefani F., Gigantiello A., Polesello S., Comandatore F., Mileto D., Maresca M., Longobardi C., Mancon A., Romeri F. (2020). Presence and infectivity of SARS-CoV-2 virus in wastewaters and rivers. Sci. Total Environ..

[26] Kitajima M., Ahmed W., Bibby K., Carducci A., Gerba C.P., Hamilton K.A., Haramoto E., Rose J.B. (2020). SARS-CoV-2 in wastewater: State of the knowledge and research needs. Sci. Total Environ..

[27] Foladori P., Cutrupi F., Segata N., Manara S., Pinto F., Malpei F., Bruni L., La Rosa G. (2020). SARS-CoV-2 from faeces to wastewater treatment: What do we know? A review. Sci. Total Environ..

[28] Wyer M.D., Wyn-Jones A.P., Kay D., Au-Yeung H.K.C., Gironés R., López-Pila J., de Roda Husman A.M., Rutjes S., Schneider O. (2012). Relationships between human adenoviruses and faecal indicator organisms in European recreational waters. Water Res..

[29] Olańczuk-Neyman K., Geneja M., Quant B., Dembińska M., Kruczalak K., Kulbat E., Kulik-Kuziemska I., Mikołajski S., Gielert M. (2003). Microbiological and biological aspects of the wastewater treatment plant “Wschód” in Gdańsk. Polish J. Environ. Stud..

[30] Koivunen J., Heinonen-Tanski H. (2005). Inactivation of enteric microorganisms with chemical disinfectants, UV irradiation and combined chemical/UV treatments. Water Res..

[31] Bodzek M., Konieczny K., Rajca M. (2019). Membranes in water and wastewater disinfection—Review. Arch. Environ. Prot..

[32] Hassen A., Mahrouk M., Ouzari H., Cherif M. (2000). UV disinfection of treated wastewater in a large-scale pilot plant and inactivation of selected bacteria in a laboratory UV device. Bioresour. Technol..

[33] Hallmich C., Gehr R. (2010). Effect of pre- and post-UV disinfection conditions on photoreactivation of fecal coliforms in wastewater effluents. Water Res..

[34] Mundy B., Kuhnel B., Hunter G., Jarnis R., Funk D., Burns N., Drago J., Nezgod W., Huang J., Jasim S. (2018). A Review of Ozone Systems Costs for Municipal Applications. Report by the Municipal Committee—IOA Pan American Group. Ozone Sci. Eng..

[35] Timm C., Luther S., Jurzik L., Hamza I.A., Kistemann T. (2016). Applying QMRA and DALY to assess health risks from river bathing. Int. J. Hyg. Environ. Health.

[36] Collivignarelli M.C. (2018). Overview of the Main Disinfection Processes for Wastewater and Drinking Water Treatment Plants. Sustainability.

[37] Furst K.E., Pecson B.M., Webber B.D., Mitch W.A. (2018). Tradeoffs between pathogen inactivation and disinfection byproduct formation during sequential chlorine and chloramine disinfection for wastewater reuse. Water Res..

[38] Lin T., Zhou D., Dong J., Jiang F., Chen W. (2016). Acute toxicity of dichloroacetonitrile (DCAN), a typical nitrogenous disinfection by-product (N-DBP), on zebrafish (Danio rerio). Ecotoxicol. Environ. Saf..

[39] Krasner S.W., Westerhoff P., Chen B., Rittmann B.E., Amy G. (2009). Occurrence of disinfection byproducts in United States wastewater treatment plant effluents. Environ. Sci. Technol..

[40] Monarca S., Feretti D., Collivignarelli C., Guzzella L., Zerbini I., Bertanza G., Pedrazzani R. (2000). The influence of different disinfectants on mutagenicity and toxicity of urban wastewater. Water Res..

[41] Sharma P.R., Sharma S.K., Lindström T., Hsiao B.S. (2020). Nanocellulose-Enabled Membranes for Water Purification: Perspectives. Adv. Sustain. Syst..

[42] Hassan M.L., Fadel S.M., Abouzeid R.E., Abou Elseoud W.S., Hassan E.A., Berglund L., Oksman K. (2020). Water purification ultrafiltration membranes using nanofibers from unbleached and bleached rice straw. Sci. Rep..

[43] Zhu C., Liu P., Mathew A.P. (2017). Self-Assembled TEMPO Cellulose Nanofibers: Graphene Oxide-Based Biohybrids for Water Purification. ACS Appl. Mater. Interfaces.

[44] Sharma P.R., Chattopadhyay A., Zhan C., Sharma S.K., Geng L., Hsiao B.S. (2018). Lead removal from water using carboxycellulose nanofibers prepared by nitro-oxidation method. Cellulose.

[45] Gholami Derami H., Gupta P., Gupta R., Rathi P., Morrissey J.J., Singamaneni S. (2020). Palladium Nanoparticle-Decorated Mesoporous Polydopamine/Bacterial Nanocellulose as a Catalytically Active Universal Dye Removal Ultrafiltration Membrane. ACS Appl. Nano Mater..

[46] Sosnowski T., Suchecka T.W.P. (2004). Penetracja komórki przez membranę mikrofiltracyjną. Proceedings of the Monografie Komitetu Inżynierii Środowiska PAN, (V Scientific Conference: Membranes and Membrane Processes in Environmental Protection, Ustroń 2004).

[47] Fujioka T., Hoang A.T., Ueyama T., Nghiem L.D. (2019). Integrity of reverse osmosis membrane for removing bacteria: New insight into bacterial passage. Environ. Sci. Water Res. Technol..

[48] Demographic Yearbook of Poland 2020, Statistics Poland. https://stat.gov.pl/en/topics/statistical-yearbooks/statistical-yearbooks/demographic-yearbook-of-poland-2020,3,14.html.

[49] PEWIK Gdynia. https://www.pewik.gdynia.pl.

[50] Czerwionka K., Makinia J., Pagilla K.R., Stensel H.D. (2012). Characteristics and fate of organic nitrogen in municipal biological nutrient removal wastewater treatment plants. Water Res..

[51] Budzińska K., Bochenek M., Traczykowski A., Szejniuk B., Pasela R., Jurek A. (2015). Eliminacja bakterii nitkowatych w osadzie czynnym pod wplywem wybranych koagulantów i związków utleniająych. Rocz. Ochr. Sr..

[52] PCI Membranes. https://www.pcimembranes.com/.

[53] APHA (2005). Standard Methods for the Examination of Water and Wastewater.

[54] Porter K.G., Feig Y.S. (1980). The use of DAPI for identifying and counting aquatic microflora. Limnol. Oceanogr..

[55] Fry J.C., Grigorova R., Norris J.R. (1990). Direct Methods and Biomass Estimation. Techniques in Microbial Ecology.

[56] Świątecki A. (1997). Zastosowanie Wskaźników Bakteriologicznych w Ocenie wód Powierzchniowych.

[57] Nübel U., Garcia-Pichel F., Kühl M., Muyzer G. (1999). Quantifying microbial diversity: Morphotypes, 16S rRNA genes, and carotenoids of oxygenic phototrophs in microbial mats. Appl. Environ. Microbiol..

[58] Norland S., Cole J.J. (1993). The relationship between biomass and volume of bacteria. Handbook of Methods in Aquatic Microbial Ecology.

[59] Chen F., Lu J.R., Binder B.J., Liu Y.C., Hodson R.E. (2001). Application of digital image analysis and flow cytometry to enumerate marine viruses stained with SYBR Gold. Appl. Environ. Microbiol..

[60] Pehlivanoglu-Mantas E., Sedlak D.L. (2008). Measurement of dissolved organic nitrogen forms in wastewater effluents: Concentrations, size distribution and NDMA formation potential. Water Res..

[61] Liu L., Smith D.S., Houweling D., Neethling J.B., Stensel H.D., Murthy S., Pramanik A., Gu A.Z. (2010). Comparison of Phosphorus Fractionation in Effluents from Different Wastewater Treatment Processes. Proc. Water Environ. Fed..

[62] Hu X., Sobotka D., Czerwionka K., Zhou Q., Xie L., Makinia J. (2018). Effects of different external carbon sources and electron acceptors on interactions between denitrification and phosphorus removal in biological nutrient removal processes. J. Zhejiang Univ. Sci. B.

[63] Kay D., Crowther J., Stapleton C.M., Wyer M.D., Fewtrell L., Edwards A., Francis C.A., McDonald A.T., Watkins J., Wilkinson J. (2008). Faecal indicator organism concentrations in sewage and treated effluents. Water Res..

[64] Koivunen J., Siitonen A., Heinonen-tanski H. (2003). Elimination of enteric bacteria in biological–chemical wastewater treatment and tertiary filtration units. Water Res..

[65] Luczkiewicz A., Fudala-Książek S., Jankowska K., Szopinska M., Björklund E., Svahn O., Garnaga-Budrė G., Tränckner J., Kaiser A. (2019). Inventory of Existing Treatment Technologies in Wastewater Treatment Plants. Case Studies in Four Coastal Regions of the South Baltic Sea. Project Model Areas for Removal of Pharmaceutical Substances in the South Baltic MORPHEUS. https://eucc-d-inline.databases.eucc-d.de/files/documents/00001203_MORPHEUS%20Del%205.1.pdf.

[66] Ikehata K., Liu Y., Sun R. (2009). Health effects associated with wastewater treatment and disposal. J. Water Environ. Res..

[67] Numberger D., Ganzert L., Zoccarato L., Mühldorfer K., Sauer S., Grossart H.P., Greenwood A.D. (2019). Characterization of bacterial communities in wastewater with enhanced taxonomic resolution by full-length 16S rRNA sequencing. Sci. Rep..

[68] Gómez M., de la Rua A., Garralón G., Plaza F., Hontoria E., Gómez M.A. (2006). Urban wastewater disinfection by filtration technologies. Desalination.

[69] Aghalari Z., Dahms H.U., Sillanpää M., Sosa-Hernandez J.E., Parra-Saldívar R. (2020). Effectiveness of wastewater treatment systems in removing microbial agents: A systematic review. Global. Health.

[70] Rizzo L., Gernjak W., Krzeminski P., Malato S., McArdell C.S., Perez J.A.S., Schaar H., Fatta-Kassinos D. (2020). Best available technologies and treatment trains to address current challenges in urban wastewater reuse for irrigation of crops in EU countries. Sci. Total Environ..

[71] Kirchman D.L., Sigda J., Kapuscinski R., Mitchell R. (1982). Statistical analysis of the direct count for enumerating bacteria. Appl. Environ. Microbiol..

[72] Bjørnsen P.K. (1986). Automatic Determination of Bacterioplankton Biomass by Image Analysis Automatic Determination of Bacterioplankton Biomass by Image Analysist. Appl. Environ. Microbiol..

[73] Shopov A., Williams S.C., Verity P.G. (2000). Improvements in image analysis and fluorescence microscopy to discriminate and enumerate bacteria and viruses in aquatic samples. Aquat. Microb. Ecol..

[74] Zeder M., Pernthaler J. (2009). Multispot Live-Image Autofocusing for High-Throughput Microscopy of Fluorescently Stained Bacteria. Cytom. Part A.

[75] Jankowska E., Jankowska K., Włodarska-Kowalczuk M. (2015). Seagrass vegetation and meiofauna enhance the bacterial abundance in the Baltic Sea sediments (Puck Bay). Environ. Sci. Pollut. Res..

[76] Górniak D., Marszałek H., Jankowska K., Dunalska J. (2016). Bacterial community succession in an Arctic lake–stream system (Brattegg Valley, SW Spitsbergen). Boreal Environ. Res..

[77] Kosek K., Kozak K., Kozioł K., Jankowska K., Chmiel S., Polkowska Ż. (2018). The interaction between bacterial abundance and selected pollutants concentration levels in an arctic catchment (southwest Spitsbergen, Svalbard). Sci. Total Environ..

[78] Kosek K., Luczkiewicz A., Kozioł K., Jankowska K., Ruman M., Polkowska Ż. (2019). Environmental characteristics of a tundra river system in Svalbard. Part 1: Bacterial abundance, community structure and nutrient levels. Sci. Total Environ..

[79] Kalinowska A., Jankowska K., Fudala-Książek S., Pierpaoli M., Luczkiewicz A. (2020). The microbial community, its biochemical potential, and the antimicrobial resistance of *Enterococcus* spp. in Arctic lakes under natural and anthropogenic impact (West Spitsbergen). Sci. Total Environ..

[80] Gaveau A., Coetsier C., Roques C., Bacchin P., Dague E., Causserand C. (2017). Bacteria transfer by deformation through microfiltration membrane. J. Memb. Sci..

[81] Krahnstöver T., Hochstrat R., Wintgens T. (2019). Comparison of methods to assess the integrity and separation efficiency of ultrafiltration membranes in wastewater reclamation processes. J. Water Process Eng..

[82] Ghuneim L.A.J., Jones D.L., Golyshin P.N., Golyshina O.V. (2018). Nano-sized and filterable bacteria and archaea: Biodiversity and function. Front. Microbiol..

[83] Noble R.T., Fuhrman J.A. (1998). Use of SYBR Green I for rapid epifluorescence counts of marine viruses and bacteria. Aquat. Microb. Ecol..

[84] Patel A., Noble R.T., Steele J.A., Schwalbach M.S., Hewson I., Fuhrman J.A. (2007). Virus and prokaryote enumeration from planktonic aquatic environments by epifluorescence microscopy with SYBR Green I. Nat. Protoc..

[85] Shibata A., Goto Y., Saito H., Kikuchi T., Toda T., Taguchi S. (2006). Comparison of SYBR Green I and SYBR Gold stains for enumerating bacteria and viruses by epifluorescence microscopy. Aquat. Microb. Ecol..

[86] Tanji Y., Mizoguchi K., Yoichi M., Morita M., Hori K., Unno H. (2002). Fate of Coliphage in a Wastewater Treatment Process. J. Biosci. Bioeng..

[87] Bunce J.T., Ndam E., Ofiteru I.D., Moore A., Graham D.W. (2018). A review of phosphorus removal technologies and their applicability to small-scale domestic wastewater treatment systems. Front. Environ. Sci..

[88] Huber M., Athanasiadis K., Helmreich B. (2020). Phosphorus removal potential at sewage treatment plants in Bavaria—A case study. Environ. Chall..

